# Hemiazygos vein dilation as a radiological finding and multifactorial cause of dysphagia

**DOI:** 10.1016/j.radcr.2023.04.040

**Published:** 2023-05-23

**Authors:** Shivam Khatri, Salomon Chamay, Steven Chacko, Shorabh Sharma

**Affiliations:** a CUNY School of Medicine, 160 Convent Ave, New York, NY 10031, USA; b Division of Internal Medicine, Department of Medicine, St. Barnabas Hospital, Bronx, NY 10457, USA

**Keywords:** Hemiazygos vein, Esophagus, Radiological scan, Gastrointestinal, Diagnostic radiology

## Abstract

Dysphagia is a common issue observed among the elderly, which can arise from various etiologies such as motility disorders and chronic neurologic conditions. Radiologists play a crucial role in diagnosing the cause of dysphagia, as they can identify anatomical abnormalities that may lead to the condition. One such anomaly is the hemiazygos vein, which is the left side equivalent of the azygos vein and can cause dysphagia if it crosses over the esophagus. To our knowledge, there are only 2 other recorded cases of azygos aneurysm/dilation causing esophageal dysphagia. In this context, we present a case report of a 73-year-old female with a 1-month history of weight loss and dysphagia due to a prominent hemiazygos vein. The case highlights the importance of thorough radiological evaluation in identifying the underlying cause of dysphagia and ensuring timely and appropriate treatment.

## Introduction

Dysphagia is a common disorder experienced by the elderly. Thirteen percent of those 65 years and older and 51% of elderly individuals in long-term care facilities are affected by dysphagia [1]. Studies report that dysphagia impacts 16% of independently living individuals aged 70-79 [2]. The most common etiologies of oropharyngeal dysphagia in this population include chronic neurologic conditions such as stroke, Parkinson disease, and dementia [3]. One study found that 32%-45% of patients with Alzheimer's disease were found to have oropharyngeal dysphagia [1]. Several physiologic changes such as loss of muscle mass and function, decreased tissue elasticity, and decreased saliva production can contribute to oropharyngeal dysphagia in the elderly [1]. In comparison, the most common causes of esophageal dysphagia include gastroesophageal reflux disease and functional esophageal disorders [3,4]. Malignancy, especially in those with associated risk factors such as smoking and alcohol, must be ruled out as a potential cause of dysphagia in the elderly.

Venous abnormalities rarely produce a mass effect, due to their easy compressibility. We report a case of a 73-year-old female with a month-long history of weight loss and dysphagia in the setting of a prominent hemiazygos vein confirmed by imaging. To our understanding, this is only the second case of a prominent hemiazygos vein causing an esophageal abnormality.

## Case presentation

A 73-year-old female with a medical history of asthma, osteoporosis, prior herniated discs, diverticulosis, prediabetes mellitus, and a 40-pack-year smoking history presented to the emergency room (ER) with a 1-month history of difficulty swallowing both fluids and solids, along with a sore throat. Four days prior to admission, she experienced one episode of black stools and right-sided neck pain while eating chicken, and reported a choking sensation, dysphagia, and odynophagia. The patient also reported night sweats, occasional constipation, sharp chest pain with swallowing, and unintentional weight loss of 10-20 pounds within the 1-month period. She denied any shortness of breath, tongue swelling, nausea, or vomiting. The patient recalled a colonoscopy 2 years prior that was inconclusive due to poor visualization from rectal bleeding.

Upon admission to the ER, the patient's vital signs were as follows: body mass index of 25, temperature of 98.0°F, blood pressure of 135/79 mmHg, heart rate of 68 bpm, and respiratory rate of 16 bpm with 98% O_2_ saturation on room air. Physical examination revealed right posterior cervical lymphadenopathy, but the patient appeared otherwise comfortable, and cardiovascular, pulmonary, and abdominal examinations were unremarkable. Palpation did not reveal any thyromegaly, and the abdomen was flat, soft, and nontender throughout. No hepatosplenomegaly was appreciated, and a digital rectal examination was negative for bright red blood or melena. Laboratory studies conducted during admission, including a complete blood count, basic metabolic panel, and hepatic function testing, were unremarkable for anemia, electrolyte, renal, or hepatic abnormalities. Given the patient's weight loss, progressive dysphagia and risk factors, esophageal malignancy was strongly considered as a potential diagnosis at this time.

**Fig. 1:**
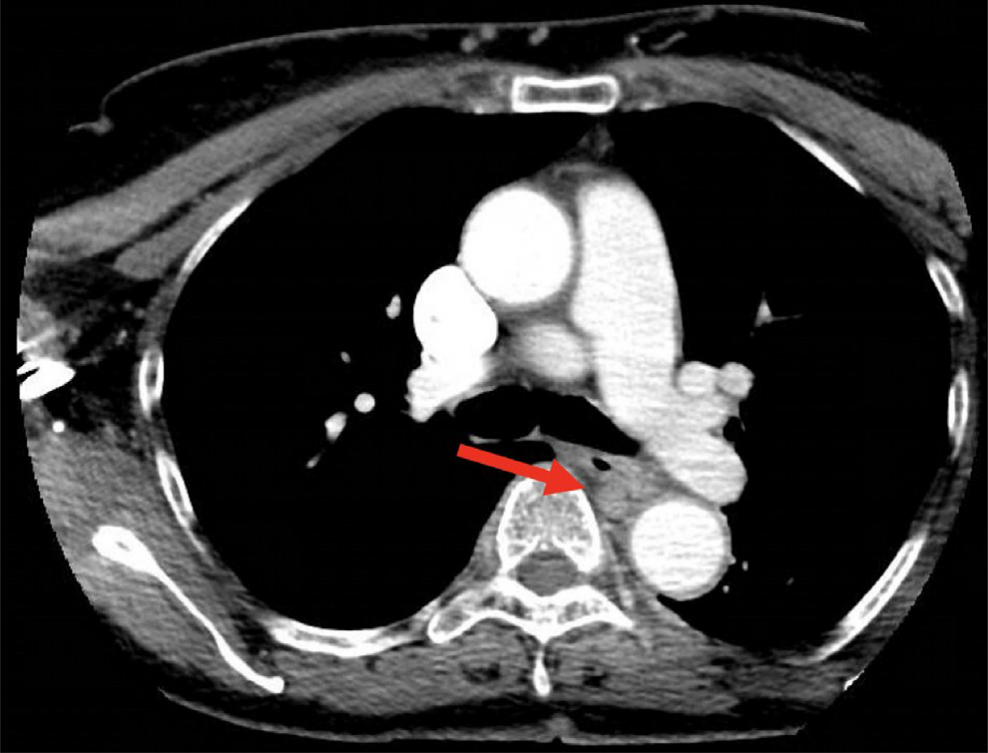
CT neck with contrast demonstrates a mass compressing the esophagus at the level of the carina.

In the ER, the patient underwent a computerized tomography (CT) scan of the neck due to symptoms of choking sensation, pain and swelling. The scan revealed mild thickening of the upper esophageal wall, and a 3.5 × 2.7 cm mass located at the level of the carina. These findings were concerning for a possible neoplasm (Fig. 1).

**Fig. 2:**
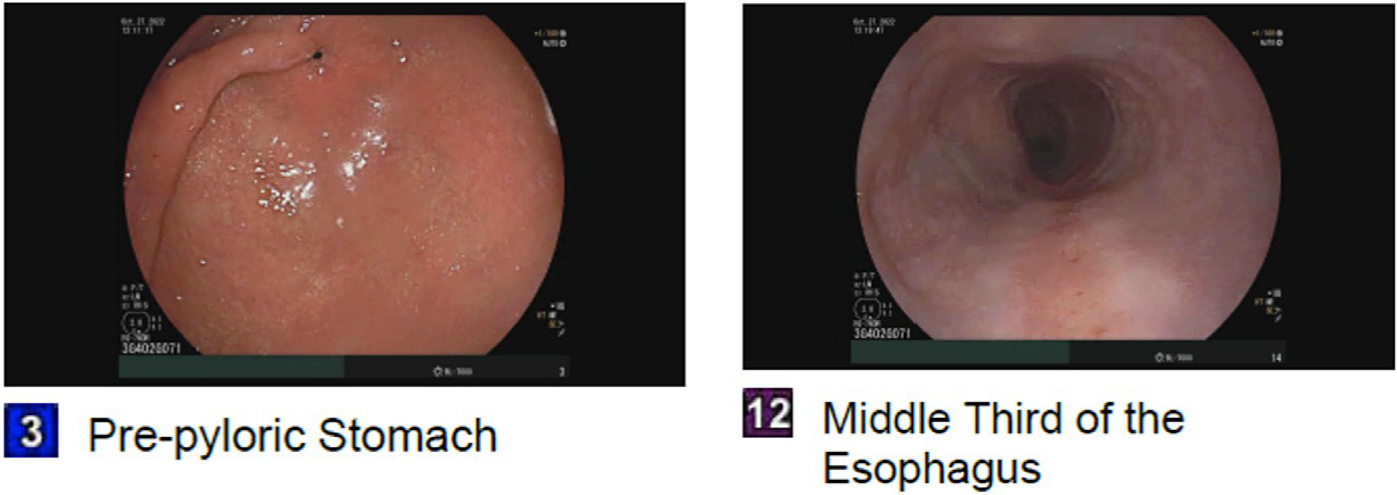
EGD. EGD, esophagogastroduodenoscopy.

After being admitted to the hospital due to a possible neoplasm, the gastroenterology service was consulted on the patient's second day of stay. They recommended an esophagogastroduodenoscopy (EGD), which was performed the same day, revealing no evidence of luminal lesions but indicating a possible superficial gastric ulcer (Fig. 2). The EGD found some findings of erythematous mucosa in the gastric body and antrum. Biopsies taken from the esophagus at the level of the mass showed normal results. As the EGD results were negative, an esophagogram was recommended to rule out potential upper motility gastrointestinal disorders. A few days later, the esophagogram was performed, which indicated no evidence of esophageal mass, achalasia, or strictures.

**Fig. 3:**
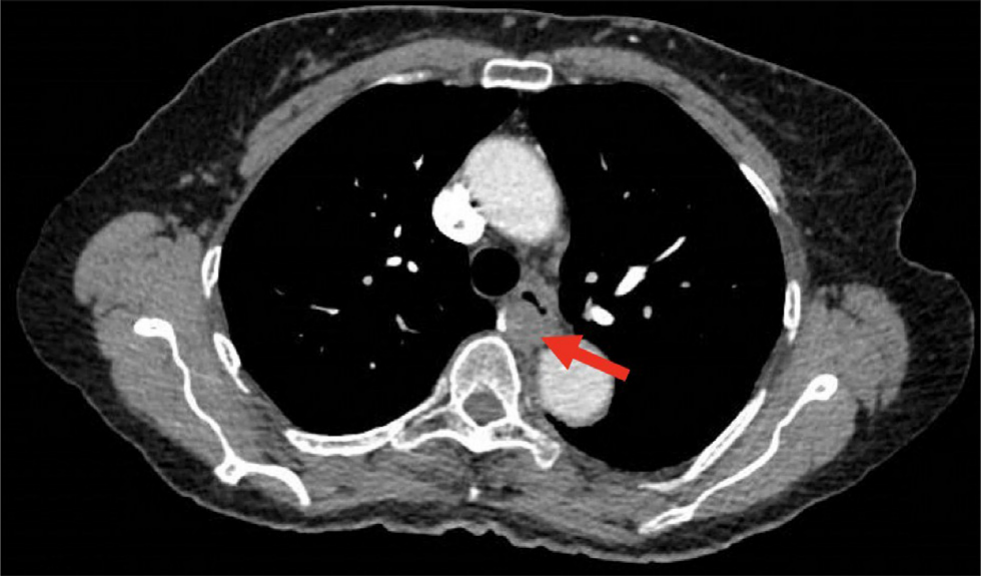
CT scan of chest demonstrating mass in posterior esophageal wall at the level of T5-T7.

**Fig. 4:**
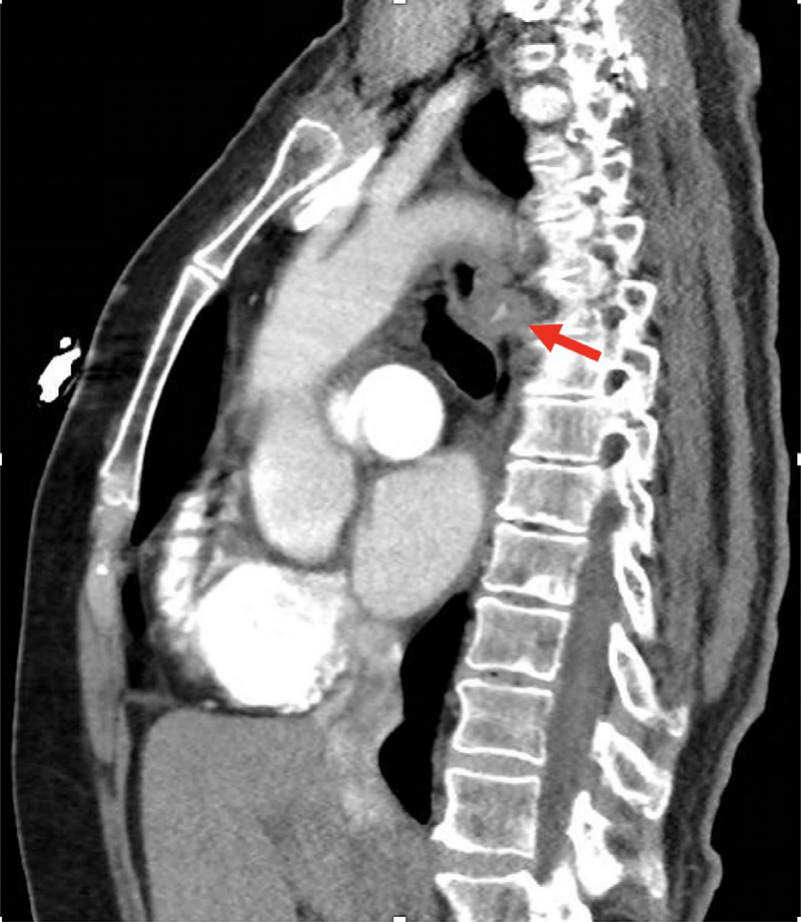
Sagittal view of CT chest with arrow pointing to a mass present in the posterior esophageal wall.

After receiving negative results from the EGD, we consulted the pulmonology service on day 7 of her stay due to the presence of right posterior cervical lymphadenopathy that was identified during the initial physical exam. Our goal was to rule out any potential intrathoracic malignancies. Following their recommendation, we conducted a CT chest with contrast, which revealed a soft tissue mass measuring 1.36 × 1.77 × 1.96 cm located in the posterior esophageal wall at the T5-T7 vertebrae level (Figs. 3 and 4).

**Fig. 5:**
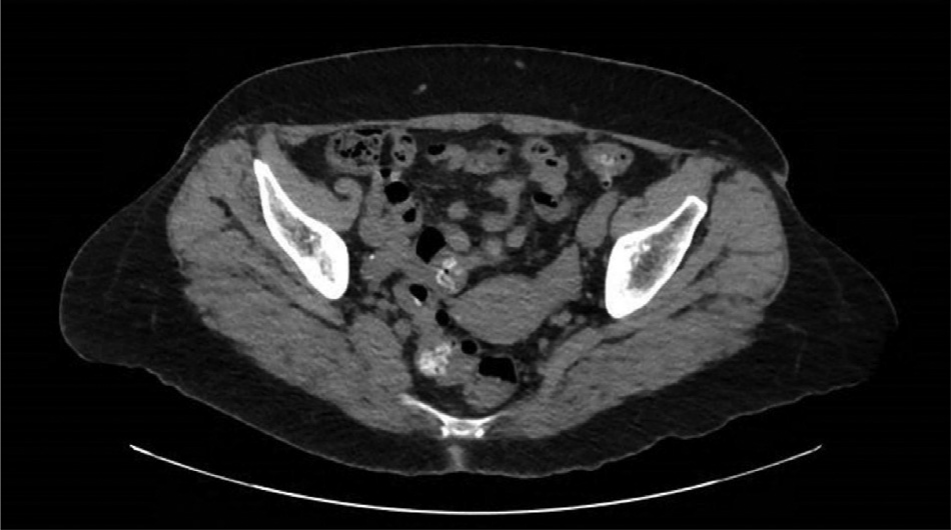
CT abdomen and pelvis.

After discovering the presence of a mass in the posterior esophageal wall, we added gastrointestinal mesenchymal tumors such as leiomyoma, leiomyosarcoma, and gastrointestinal stromal tumor to our list of potential diagnoses due to the possibility of involvement of the submucosal and muscularis layers of the esophagus. To gain further insight, we ordered a CT abdomen and pelvis, which revealed scattered diverticulosis of the colon consistent with previous CT scan findings (Fig. 5). Thickening and edema of the pyloric antral wall was also noted, which possibly indicated gastritis.

**Fig. 6:**
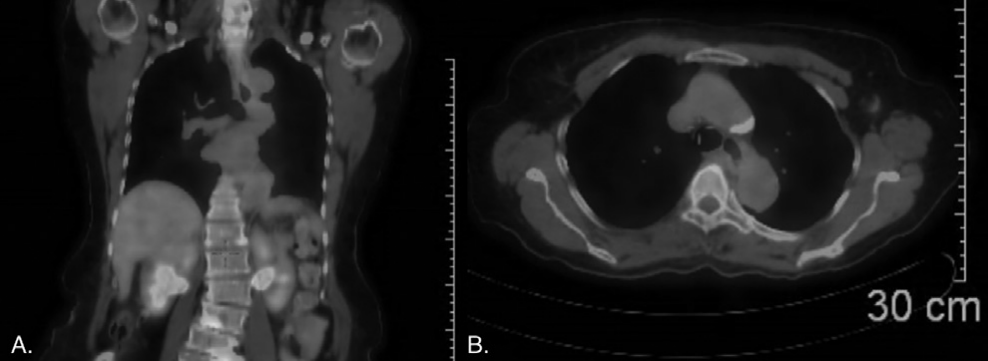
(A) Coronal view of positron emission tomography (PET) scan. (B) Axial view.

On the eighth day of hospitalization, the interventional radiologist recommended a positron emission tomography (PET) scan after reviewing the CT images mentioned above to conclusively rule out the possibility of a pathological mass, and also recommended an endoscopic ultrasound. Upon review with radiology, it was discovered that a mass next to the mid-thoracic esophagus, initially detected by the PET scan, was actually a prominent hemiazygos vein with no significant increase in metabolic activity (Fig. 6).

Based on the PET scan result, negative esophageal biopsies, a mass in the posterior esophageal walls, and negative esophagogram and EGD, malignancy was considered unlikely, and a diagnosis of hemiazygos vein causing esophageal compression with dysphagia was made. The patient was advised to follow up with an endoscopy in 3 months, CT scan of the abdomen/pelvis in 6 months, and a 12-week course of proton pump inhibitors twice a day. The patient was asked to follow up in the outpatient clinic; however, was lost to follow-up.

## Discussion

Although there are approximately 50 case reports of azygos vein aneurysm (AVA), only one mentions the dilation of its tributary vein, the hemiazygos vein [5]. Moreover, AVA is often asymptomatic and largely an incidental finding, which limits the information available on its management and diagnosis [6].

The cause of hemiazygos vein dilation can be further classified into physiologic and pathologic etiologies. Physiologic causes include azygos continuation of the inferior vena cava (IVC), and congenital superior vena cava (SVC) or IVC interruptions. In contrast, pathologic causes of a dilated hemiazygos include congestive heart failure, constrictive pericarditis, SVC and IVC obstruction, pericardial effusion, portal hypertension, right ventricular strain, and tricuspid insufficiency [6,7] However, most cases of hemiazygos vein dilation are idiopathic in nature and a cause is not identified [6], [7], [8]. In this case, our patient had a dilated hemiazygos of unknown etiology as she had no past medical history of congestive heart failure, and no signs of IVC obstruction, portal hypertension, right ventricular strain, or signs of congenital abnormalities related to the hemiazygos vein.

To understand the potential cause of dysphagia in this patient due to hemiazygos vein dilation, it is essential to comprehend the anatomy of the azygos vein. The azygos vein originates from the confluence of the upper lumbar and right subcostal veins before entering the thorax [8]. Consequently, it runs in close proximity to the esophagus and thoracic duct through the posterior mediastinum and receives contributions from the hemiazygos vein and accessory hemiazygos vein [8,9]. At the level of T8-T9, the hemiazygos vein enters the azygos vein and drains into the SVC [10]. Since the azygos vein arches over the esophagus anteriorly before its termination in the SVC, any dilation or aneurysm in the vein has the potential to cause dysphagia symptoms, as in this patient [7].

As mentioned earlier, the majority of cases of hemiazygos vein dilation or AVA are often asymptomatic and are discovered incidentally. However, symptoms may arise when aneurysms are larger than 5 cm and compress surrounding structures such as the esophagus, bronchi, SVC, and pneumogastric nerve [6]. According to Newton et al. [11] symptomatic patients may experience chest tightness, dysphagia, palpitations, or coughing. Savu et al. [6] also mention that patients with AVA may experience heart palpitations, wheezing, and persistent hiccups.

To better understand the presentation of hemiazygos vein dilation/AVA, we can look at other case reports. Currently, to our knowledge, only 2 other recorded cases of azygos aneurysm/dilation causing esophageal dysphagia exist. Morton et al. [8] describe a case of a patient with a large aneurysm of the azygos vein near the SVC causing intermittent dysphagia, which was treated with surgical myotomy and partial fundoplication. Our case is similar, given the finding of an enlarged hemiazygos vein compressing the esophagus and presenting as dysphagia. Odedra et al. [10] reported a case of a 59-year-old Indian woman with a history of deep vein thrombosis and gastric ulcer diagnosed with EGD, who presented to the respiratory clinic with chest pain, dysphagia, and an abnormal chest radiograph. An eventual MRI confirmed the absence of mediastinal lymphadenopathy and the presence of a prominent hemiazygos vein compressing the esophagus, which is similar to the presentation in this case. In this case, the patient was also started on proton-pump therapy for resolution of symptoms [10].

Although complications such as mass effect have been described as a result of AVA/hemiazygos vein dilation, there is no universally accepted management strategy. For asymptomatic patients, a conservative or surveillance approach is utilized in the majority of cases [8,12]. For symptomatic patients, one approach encompasses the prevention of thromboembolism and rupture when an aneurysm is involved as well as the alleviation of symptoms [8]. In this case, close observation and symptomatic relief with proton pump inhibitors was the initial choice. Surgical resection with or without venous bypass has also been performed, and yielded excellent results [8,12]. With the advent of endovascular approaches, stenting, venous occlusion, and embolization have been suggested as safe and effective ways to repair AVA/hemiazygos vein dilation [8]. Stenting is favored due to its ability to exclude the aneurysm while preserving venous patency; however, this option may be limited by anatomic location. Perhaps with increased knowledge about AVA, multidisciplinary teams will be able to better come up with approaches to treat AVA, whether surgical or symptomatically.

## Conclusion

When elderly patients present with dysphagia, consideration of other multifactorial causes of dysphagia is important, in addition to more common etiologies. In cases of hemiazygos vein dilation, radiologists play a crucial role in detecting and diagnosing the condition using imaging such as CT, MRI, PET, and ultrasound. Once diagnosed, surgeons may be consulted to determine the appropriate management strategy, whether it be conservative observation or surgical intervention.

## Author Contributions

**Shivam Khatri:** Case Writer and Literature Review. **Salomon Chamay:** Case Writer and Literature Review **Steven Chacko:** Case Writer and Literature Review. **Shorabh Sharma:** Editor.

## Patient consent

Informed consent was obtained from the patient before writing up this case study.
